# Sodium Montmorillonite/Amine-Containing Drugs Complexes: New Insights on Intercalated Drugs Arrangement into Layered Carrier Material

**DOI:** 10.1371/journal.pone.0121110

**Published:** 2015-03-24

**Authors:** Murilo L. Bello, Aridio M. Junior, Bárbara A. Vieira, Luiza R. S. Dias, Valéria P. de Sousa, Helena C. Castro, Carlos R. Rodrigues, Lucio M. Cabral

**Affiliations:** 1 Laboratório de Modelagem Molecular e QSAR (ModMolQSAR), Faculdade de Farmácia, Universidade Federal do Rio de Janeiro, Rio de Janeiro, RJ, Brazil; 2 Laboratório de Tecnologia Farmacêutica Industrial (LabTIF), Faculdade de Farmácia, Universidade Federal do Rio de Janeiro, Rio de Janeiro, RJ, Brazil; 3 Laboratório de Química Medicinal (LQMed), Faculdade de Farmácia, Universidade Federal Fluminense, RJ, Brazil; 4 LABiEMol, Instituto de Biologia, Universidade Federal Fluminense, Niterói, RJ, Brazil; Jacobs University Bremen, GERMANY

## Abstract

Layered drug delivery carriers are current targets of nanotechnology studies since they are able to accommodate pharmacologically active substances and are effective at modulating drug release. Sodium montmorillonite (Na-MMT) is a clay that has suitable properties for developing new pharmaceutical materials due to its high degree of surface area and high capacity for cation exchange. Therefore Na-MMT is a versatile material for the preparation of new drug delivery systems, especially for slow release of protonable drugs. Herein, we describe the intercalation of several amine-containing drugs with Na-MMT so we can derive a better understanding of how these drugs molecules interact with and distribute throughout the Na-MMT interlayer space. Therefore, for this purpose nine sodium montmorillonite/amine-containing drugs complexes (Na-MMT/drug) were prepared and characterized. In addition, the physicochemical properties of the drugs molecules in combination with different experimental conditions were assessed to determine how these factors influenced experimental outcomes (e.g. increase of the interlayer spacing versus drugs arrangement and orientation). We also performed a molecular modeling study of these amine-containing drugs associated with different Na-MMT/drug complex models to analyze the orientation and arrangement of the drugs molecules in the complexes studied. Six amine-containing drugs (rivastigmine, doxazosin, 5-fluorouracil, chlorhexidine, dapsone, nystatin) were found to successfully intercalate Na-MMT. These findings provide important insights on the interlayer aspect of the molecular systems formed and may contribute to produce more efficient drug delivery nanosystems.

## Introduction

Clay minerals are abundant raw materials that possess a variety of physical-chemical properties [1, 2]. They have a range of uses in the pharmaceutical industry due to their structural properties and composition. In addition, clay minerals have excellent adsorption properties, which can be attributed to their large surface area. This property makes them important candidates for adsorption or fixation of drug molecules, polymers, virus, bacteria and other substances to their surface [3, 4]. Since clay minerals are inexpensive, biocompatible and possess favorable thermal stability, they are ideal for use as drug carriers [5–7]. According to published studies, a promising alternative approach for the production of new modified release and drug delivery systems can be obtained by combining porous mineral clays with pharmacologically active organic molecules [8, 9]. Sodium montmorillonite (Na-MMT) is the most often used layered silicate in the pharmaceutical technology research field [10]. Na-MMT has a 2:1 stacked structure, consisting of two tetrahedral sheets and one octahedral sheet. This clay mineral has a negative charge profile generated by the isomorphic substitution of aluminum (Al) by magnesium (Mg) ions and is further stabilized by Na^+^ cations [11]. Moreover, the interaction of Na-MMT with organic substances such as drugs or polymers can generate intercalated or exfoliated materials by substitution of sodium cations in the clay interlayer space or by surface adsorption [12]. This reaction depends on the physical-chemical properties of the molecule that will be inserted into the lamellar silicate carrier. The formation of intercalated or exfoliated nanosystems is dependent on several factors including experimental conditions and the type of organic material and layered silicate that are used [13].

To further assess the potential use of Na-MMT as a drug carrier, especially for amino molecules, it is important to use complementary techniques. On this context, molecular modeling provides a good platform for better understanding the intercalation mechanisms and thus, to contribute for the development of new drug delivery systems [14, 15]. Atomistic molecular dynamics simulation is a very useful tool that allows information to be obtained regarding conformational rearrangements of compounds as well as assessing interactions that occur amongst the different components of a molecular system. This simulation tool provides insight into molecular systems that are not easily measured using experimental methods [16, 17].

The aim of this study was to perform and analyze the intercalation reaction of nine different amine drugs with Na-MMT to generate different Na-MMT/drug complexes. In addition, we also applied a computational methodology to further assist in understanding the intercalation of organic amine-containing compounds as well as their arrangement in Na-MMT in the dry state. Overall, this study was designed to develop improved nanostructured systems using Na-MMT for drug delivery.

## Materials and Methods

### Materials

Na-MMT was purchased from ACROS (New Orleans, USA). The drug compounds utilized in this study were obtained from the following vendors: 5-fluorouracil (5FU) (Sigma-Aldrich; São Paulo, Brazil), acyclovir (ACV) (Ciel Pharmaceuticals; Coimbatore, India), chlorhexidine (CLX) gluconate (Henrifarma; São Paulo, Brazil), dapsone (DPS) (G. Alphalab; Bangalore, India), doxazosin (DXZ) mesilate (Fagron; São Paulo, Brazil), nystatin (NTT) (Genix Industria Farmacêutica; São Paulo, Brazil), rivastigmine (RVT) (Zhejian Jiuzhou Pharmaceutical Co.; Taizhou, China), and octyl dimethyl PABA (4-aminobenzoic acid) (ODP) and 2-phenylbenzimidazol-5-sulfonic acid (PSA) were purchased from Merck (Darmstadt, Germany). Hydrochloric acid was purchased from Tedia (Fairfield, USA). Acetic acid and sodium hydroxide (P.A.) grade were acquired from Vetec (Rio de Janeiro, Brazil).

### Sodium Montmorillonite Inclusion Complex Preparation

Initially, the preparation of inclusion complexes involved exploration of the maximum Na-MMT cation exchange capacity (CEC) (100 meqv of cation/100 g of Na-MMT). The mass relation of different materials was found when dispersed in a 200 ml volume of various conditions: acidic (HCl or acetic acid), basic or pure distilled water and stirred during the reaction time with a magnetic stirrer at room temperature. Subsequently, the material was centrifuged at 4000 rpm for 40 minutes. The supernatant was filtered with a 0.45 μM membrane filter to remove suspended particles and the remaining molecules were quantified using a UV-Visible spectrophotometer Thermo Fischer Scientific Genesys 10UV. The sediment was vacuum dried using a vacuum and the obtained solid was triturated before characterization. Different experimental conditions were used to test the inclusion properties and activities of Na-MMT, and are described in Table 1.

**Table 1:** Experimental conditions applied in Na-MMT/amine-containing drugs intercalation.

Systems	Drug/Na-MMT ration (mg/g)	Solvent	pH	Reaction time	Wavelength (nm)*
Na-MMT/RVT	100	Water	∼5.0	0.5h	215
Na-MMT/DXZ	80	Water	∼5.0	18h	240
Na-MMT/5FU	80	Water	∼5.0	24h	265
Na-MMT/CLX	60	Water	∼5.0	24h	240
Na-MMT/ODP	440	Acetic acid:water 2:1	∼0.8	0.5h	311
Na-MMT/ACV	444	HCl 0.1N	∼2.0	0.5h	270
Na-MMT/DPS	60	HCl 0.1N	∼2.0	24h	270
Na-MMT/NTT	60	Acetic acid 0.9%	∼2.8	24h	279
Na-MMT/PSA	365	NaOH 0.02N	∼11.0	2h	302

*Wavelength used for intercalation yield determination.

### Characterization of the Sodium Montmorillonite Inclusion Complex

Na-MMT inclusion complexes were characterized by X-ray powder diffraction (XRPD), Fourier Transform Infrared (FT-IR), differential scanning calorimetry (DSC) and thermogravimetric analysis (TGA). XRPD patterns were obtained using a Rigaku Miniflex diffractometer (Japan) with a CuKα radiation source and current of 30mA, voltage of 40kV and 2θ angle between 2° to 20°. The FT-IR spectra were obtained using an IR Prestige-21 Shimadzu spectrometer (Japan), 1.0% w/w KBr pellets and a wavelength between 4000 and 400 com^-1^. DSC analysis was performed on a DSC 60 Shimadzu thermal analyzer (Japan) using hermetically sealed aluminum pans and nitrogen flow set at 50 mL.min^-1^ and heating rate of 10°C.min^-1^ ranging between 25° to 250°C. Additionally, TGA analysis was performed using a TA-60WS Thermal Analyser Shimadzu (Japan). The samples were heated over a range between 25° to 700°C, under a 50 mL.min^-1^ nitrogen flow and the heating rate was 10°C.min^-1^.

### Computational Simulation and Molecular Modeling

#### Amine-Containing Drugs Molecular Modeling

Assessment of the distribution of possible conformations was performed under vacuum for all nonprotonated and protonated/deprotonated drug structures within a mechanical molecular force field MMFF [18]. From these simulations, the conformer with the lowest energy state was chosen using the following steps [19]. These energy minimized drug structures were first subjected to an equilibrium geometry calculation using the semi-empirical RM1 method [20]. Then a single-point calculation using the density function theory (DFT) method with functional B3LYP and 6–31G* quantum base was performed to evaluate their electronic properties. All of the above steps were carried out using the Spartan’10 V.1.1.0 program (Wavefunction Inc. Irvine, CA, 2000). Models of optimized drug molecules were used to build the various Na-MMT/drug molecular systems in the Materials Studio program package (v. 4.3 Accelrys, San Diego, CA). The values of pKa and lipophilicity (LogP and LogD) of the drugs were obtained using the ChemAxon software available at http://www.chemicalize.org. Also included was the population of the compound microspecies and the chemical structural functional group that undergoes protonation or deprotonation according to the approximated experimental pH [21].

#### Na-MMT/Amine-Containing Drug Models Build

A reliable structural model of Na-MMT was constructed for use in computer simulation studies. This model was made using the Crystal Builder module from Materials Studio molecular modeling package (v. 4.3 Accelrys, San Diego, CA) according to crystallographic coordinates from experimentally obtained data available in the literature [22]. This step involved building the MMT crystal unit cell, followed by formation of the lamellar structure. The chemical structure of MMT is characterized by random isomorphic substitution of aluminum (Al) by magnesium (Mg) atoms. These minerals form layers which consist of arrays of octahedral aluminates (AlO_6_) that exist between two arrays of tetrahedral silicates (SiO_4_) [23]. The crystal unit cell was replicated three times at the *a* axis and two times at *b* axis, therefore obtaining a cell with the following dimension 15.6 Å x 18.4 Å x 10.13 Å (unit cell dimensions was 5.20 Å x 9.20 Å x 10.13 Å). After construction of the cell, the four aluminum atoms were substituted by an equivalent number of Mg atoms, resulting in a MMT model with the chemical formula (Al_3.33_Mg_0.67_)Si_8_O_20_(OH)_4_-(Na_0.67_), as described by Scocchi and coworkers (2007) [24]. The charge defect caused by isomorphic substitution of Mg was compensated by the insertion of four Na^+^ atoms. The partial charges were ascribed to each atom according to the methods proposed by Heinz and coworkers (2005), in order to generate a structural model of a neutrally charged Na-MMT that can be used for simulations [25]. In order to construct the Na-MMT/drug molecular systems in the Materials Studio package, available optimized drug molecule models were used. The cell containing the drug molecules were systematically generated of equal dimension to the Na-MMT crystal periodic boundary. This procedure was important for maintaining Na-MMT in the correct dimension (*a* x *b* x *c*) and generating a reliable Na-MMT/drug system. PCFF_phyllosilicates were used as a standard, to assure accuracy and the appropriate force field parameters for all atoms contained in the molecular system [25]. Thereafter, all Na-MMT/drug complex models were formed using the Layer Builder module of the Materials Studio package and then submitted to computational simulations.

#### Molecular Dynamics Simulations

Prior to performing the molecular dynamic simulations, the Na-MMT/drug complexes were minimized and optimized using the Conjugate Gradient method [26]. This method provides a customized convergence level and adequately prepares the molecular system for simulation. The first simulation was conducted using a NVT ensemble at 298 K for 500 ps which equilibrated the cell in preparation for the properties calculation. The simulations were performed in this manner; an NPT ensemble simulation was run at 298 K for 1 ns using the Ewald summation method that provided a convention for non-bond electrostatic interactions [27]; an integration time step of 1 fs was utilized. During these simulation steps the Berendsen thermostat was also utilized and the pressure was kept at 1 atm [28]. The simulations were carried out using the Materials Studio Discover module with the PCFF_phyllosilicates force field and the charge scheme previously described by Heinz and coworkers (2005). Additional criteria were applied, including no movement/positional constraint of atoms in the system. The results of molecular dynamics simulations were analyzed to observe the molecular arrangements of drugs molecules in order to correlate them with the experimental basal spacing. The interlayer spacing of the models of the Na-MMT/drugs systems were analyzed every 50 ps of molecular dynamics in order to obtain the average basal spacing of the molecular systems models. We calculated the interaction energy E_interaction_ in the solid state as the non-bond intermolecular interaction energy using the following equation [29, 30];

$$E_{interaction}\;=\;E_{total}–\left(E_{MMT}\;+\;E_{drug}\right)$$ (1)

where E_total_ is the total potential energy of the binary system (Layer1-drug molecule, Layer2-drug molecule) in the Na-MMT/drug complex; E_MMT_ is the total potential energy of the MMT layer and E_drug_ is the total potential energy of the drug in the system. E_MMT_ is the energy of MMT layer in the absence of drug and the Na^+^, and E_drug_ is the energy of the isolated drug (absence of MMT). These single point energies calculations were performed on snapshots during the last 300 ps of the trajectories in order to obtain the average interaction energies of the drugs and layer within the Na-MMT/amine-containing drug systems [31]. All simulations were performed in the dry state.

## Results and Discussion

Despite of the adsorption and intercalation properties of Na-MMT have been extensively described in the literature [1, 2, 5, 11, 15], little information is available regarding how amine-containing drugs are arranged in the interlayer space of Na-MMT. In this study we analyzed the intercalation of amine-containing drugs in Na-MMT using experimental assays and molecular dynamics simulations. This approach allowed us to correlate the molecular orientation of amine-containing drugs with the amount of basal spacing found in Na-MMT.

### Amine-Containing Drugs Intercalation into Na-MMT

**Fig 1:**
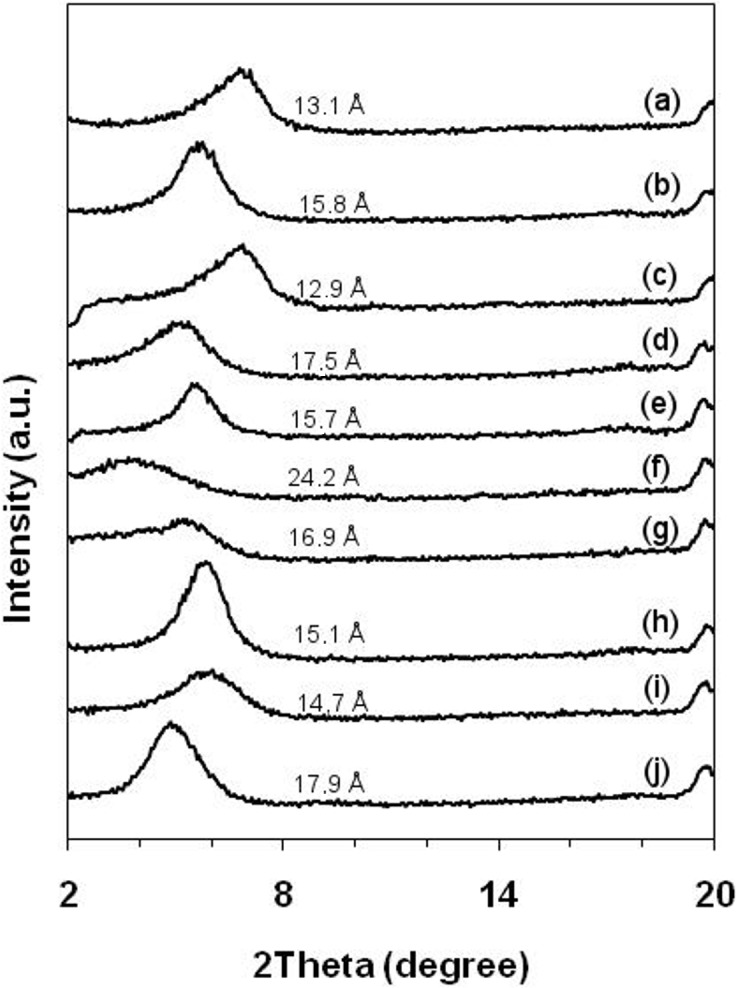
XRPD patterns 2θ of amine drugs intercalated in the montmorillonite. Sodium montmorillonite (a); 5-fluorouracil—5FU complex (b); Acyclovir—ACV complex (c); Chlorhexidine—CLX complex (d); Dapsone—DPS complex (e); Doxazosin—DXZ complex (f); Nystatin—NTT complex (g); Octyl dimethyl PABA—ODP complex (h); 2-phenylbenzimidazol-5-sulfonic acid—PSA complex (i); Rivastigmine—RVT complex (j).

Characterization of the basal spacing in the Na-MMT was performed by XRPD analysis for all of the different Na-MMT/drug complexes included in this study (Fig. 1).

The observed decrease in the 2θ value found by XRPD analysis is indicative of intercalation and is associated with increased basal spacing that occurs as a result of drug intercalation [32]. Our results indicated that almost all of the Na-MMT/drug complexes studied here had a 2θ value decrease when compared to pure sodium montmorillonite (Fig. 1). This finding provides evidence that the intercalation process was successful since most of the Na-MMT/drug complexes produced dispersions in water. However, upon stirring, the Na-MMT/RVT complex developed a gel-like consistency. In contrast to other drugs, this result indicates that RVT alone was able to partially exfoliate or disaggregate Na-MMT, leading to the formation of colloidal particles and a gelled reaction medium. This suggest that preferred molecular orientation of RVT may be related to the partially exfoliation of Na-MMT.

By variation of pH, can happen the protonation or deprotonation of the clay edges [33]. At low pH ~2 as shown in Table 1, some protonation of the clay edges (AlOOH to AlOOH_2_^+^X^-^) may occur, increasing the adsorbed amount of drugs on MMT surface. This can be the case of ODP, DPS and NTT which showed drug loaded amount at acidic media. On the other hand, at high pH ~11 for PSA intercalation experiment some deprotonation of edge groups may occur, leading to some negative charges (AlOOH to AlOO^-^Na^+^) that may have affected the adsorption of deprotonated PSA molecules on the MMT surface.

The interlayer spacing of the Na-MMT/drug complexes and Na-MMT alone was calculated using Bragg’s equation. Na-MMT exhibited a (001) diffraction maximum at 2θ of 6.75°, with a corresponding basal spacing (d_001_) of 13.1 Å. The interlayer space of the Na-MMT inclusion complex showed increased basal spacing (observed as a 2θ decrease in value). This increase in basal spacing was observed for all systems except for the ACV inclusion complex which had an interlayer spacing of 12.9 Å. The observed reduction of the interlayer space in the presence of ACV indicated that the clay was partially sealed and that intercalation had not occurred. Furthermore, it was observed that the amount of ACV drug loaded into Na-MMT was close to zero, which suggests again that there was no intercalated drug in the system. Among all complexes, Na-MMT/DXZ exhibited the lowest 2θ value, which corresponded to the highest interlayer spacing value (24.2 Å). At same time, the Na-MMT/DXZ complex exhibited the highest intercalation yield (247.2 mg/g), which provides strong evidence that the drug had intercalated abundantly into the clay. Table 2 summarizes the d_001_ values and the amount of drug-loaded MMT complex obtained.

**Table 2:** Drug-loaded amount, and experimental and simulate basal spacing of Na-MMT/amine-containing drugs complexes.

System	Drug-loaded amount/ MMT (mg/g)	Experimental basal spacing (Å)	Simulate basal spacing (Å)**
Na-MMT/RVT	114.0	17.9	17.62
Na-MMT/DXZ	247.2	24.2	23.98
Na-MMT/5FU	89.2	15.8	15.79
Na-MMT/CLX	175.5	17.5	18.07
Na-MMT/ODP	257.5	15.2	-
Na-MMT/ACV	-	12.9*	-
Na-MMT/DPS	110.7	15.7	15.55
Na-MMT/NTT	230.7	16.9	16.51
Na-MMT/PSA	-	14.7*	-

*No intercalation evident.

**Basal spacing results of the models closer to experimental results. Average basal spacing in 1 ns of molecular dynamics simulations.

The slight increase in interlayer spacing observed for the Na-MMT/PSA complex was likely due to the insertion of solvent into the Na-MMT structure or a hysteresis processes but no sealing had occurred. This scenario is predicted since the amount of drug intercalated into the system was close to zero. Additional inclusion complexes such as Na-MMT/5FU increased the interlamellar spacing and lowered the intercalation yield for Na-MMT. The amine-containing drugs ODP, DXZ and NTT led to the highest drug-loaded amount into Na-MMT, 257.5, 247.2 and 230.7 mg/g respectively. However, Na-MMT/ODP complex showed a slight increase in interlayer spacing which may be due the low intercalation and high adsorption on MMT lamellar surface.

The compounds DXZ, RVT and CLX also demonstrated the largest increase in interlamellar spacing, indicating that drugs containing aliphatic amines are more efficient at intercalating Na-MMT. Additional characterization was performed using the FT-IR and DSC techniques to confirm whether Na-MMT/drug complexes formed after XRPD characterization. Analysis of the FT-IR spectra visually identified major bands of the drugs and MMT. In addition, this technique allowed evaluation of the degree of overlap between leading spectral bands of the drug and major bands present in the Na-MMT spectra. Evaluation of the DSC curves indicated that the endothermic peak occurred at 100°C, which can be attributed to loss of water and lack of thermal events associated with formation of Na-MMT/drug complexes. No significant shifts occurred in the FT-IR bands of all the complexes produced in this study. Thermal properties of the complex materials were evaluated by TGA analysis and thermal decomposition curves indicated that a loss of sample weight occurred around 100°C, likely due to evaporation of water and degradation of intercalated drugs. In addition, the sharp weight loss suggested the possibility that the drugs formed inclusion complexes that had increased thermal stability [34].

### Investigation of the Na-MMT/Amine-Containing Drug Complexes Formation

It is known that intercalation is a relatively complex process, which depends on a number of factors associated with the physical-chemical characteristics of Na-MMT. This includes individual properties of the compounds and differences arising from the experimental conditions. Our experimental results indicated that amino-based drugs have affinity for the interlayer space of Na-MMT except for ACV and PSA. ODP molecules probably remained adsorbed on the lamellar surface, but intercalated low quantity with Na-MMT. Therefore, we applied molecular modeling and computer simulations in order to analyze the drugs intercalated in Na-MMT. Table 1 shows the experimental pH conditions whereas Table 3 displays the prevalent drugs molecules forms in this environment including: dipole moment, lipophilicity (LogP and LogD), hydrogen bonding donor groups (HBD) and hydrogen bonding acceptors groups (HBA) as well as drug volume.

**Table 3:** Physical-chemical properties of prevalent amine-containing drugs state at the intercalation pH.

Drug	Non-ionized/ionized					
	Dipole^*a*^	LogP	LogD	HBD	HBA	Volume (Å^3^)
RVT^*c*^	9.49	−1.09	−0.94	1	4	257.48
DXZ^*c*^	13.88	0.31	0.43	2	10	394.36
5FU^*b*^	3.84	−0.66	−0.66	2	4	93.61
CLX^*c*^	18.53	−8.48	−4.92	10	10	446.41
ODP^*c*^	20.72	2.04	3.20	1	3	296.10
ACV^*c*^	9.05	−2.03	−1.61	4	8	186.15
DPS^*c*^	18.63	−0.97	0.63	2	5	213.85
NTT^*c*^	15.35	−3.44	−3.00	12	18	886.80
PSA^*d*^	14.29	−0.82	−0.11	1	6	214.93

^*a*^Dipole moment in debye (D)

^*b*^non-ionized drug

^*c*^protonated drug

^*d*^deprotonated drug.

In order to obtain information on drugs prevalent forms (nonprotonated, protonated and deprotonated), we used the Chemicalize program and analyzed the occurrence of these populations based on experimental pH values during the intercalation process. The main state of each drug used to build and model the subsequent molecular dynamics study is summarized in Table 3. These data are crucial since compound protonation plays a significant role in cation exchange processes and exchange of sodium ions of Na-MMT [35]. Unlike other drugs, PSA yielded theoretically deprotonated compounds, whereas 5FU showed more concentration of non-ionized forms. This result suggests that the charge density may dictate in some cases whether nonprotonated compounds can be adsorbed to the interlayer space of Na-MMT. Furthermore, deprotonated PSA had lost a hydrogen bond donor group, important for dipole-dipole interactions with the surface of Na-MMT. This finding is in accordance with Bongur et al. (2010) that reported that the sulfonic acid group of PSA is deprotonated at basic pH [36].

The compound ACV is the only protonated drug that did not showed drug-loaded amount. Similar to DXZ, the favorable ACV protonation occurred on its structural ring moiety. However the experiments with both drugs were performed in different solvents. The results for DXZ suggest that it had intercalated to a high degree and had successfully increased the interlayer space (Table 2). The intercalation of ACV in Na-MMT can have been hindered due to competition between H_3_O^+^ in the HCl media and ACV for surface interactions with MMT [37]. Choi et al. (2004) showed that the high dipole moment found in monomers is associated with expansion of basal spacing in MMT [38]. The analysis of the dipole moment for ACV showed the lowest value (9.05 D) among the protonated forms of the amine-containing drugs (Table 3). This suggests that protonation of the ACV ring system did not greatly alter the dipole moment compared to other amine-containing drugs.

The non-ionized drug 5FU showed a small dipole moment (3.84 D) (Table 3), presenting lowest drug-loaded quantity (Table 2), but with evidence of intercalation with Na-MMT. The low volume (93.61 Å^3^) can facilitate entry of 5FU into the interlayer space since this experiment was performed in water. The protonated ODP molecules showed high drug-loaded quantity with Na-MMT, but with low basal spacing results, pointing to the low intercalation quantity with Na-MMT main due the higher ODP molecules lipophilicity (Table 3). Furthermore, the experiments performed in water yielded complexes with larger basal spacing (Na-MMT/RVT, Na-MMT/DXZ and Na-MMT/CLX). This finding indicates the importance of water in the Na-MMT swelling process.

**Fig 2:**
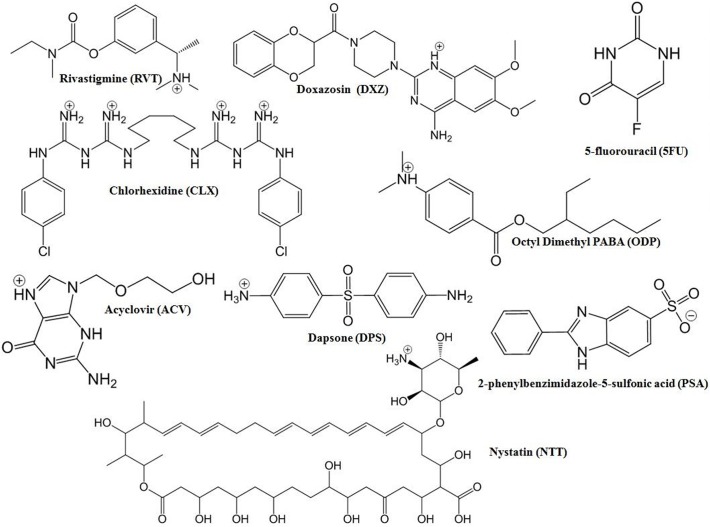
Predominant structural forms of amine drugs in experimental conditions applied during the intercalation procedure. The predictions of the prevalent forms were obtained using ChemAxon software available at http://www.chemicalize.org.

Intercalation does not occur exclusively by cationic bonding (cation exchange), in which protonated amine compounds replace sodium ions present in MMT layers. Additional mechanisms lead to intercalation including adsorption due to ion-dipole interactions, dipole-dipole interactions and hydrogen bonding between polar organic molecules and hydroxyl groups or oxygen in the Na-MMT layer [38]. The compounds RVT, DXZ, CLX, DPS and NTT showed protonated species in the experimental conditions used in this study (Table 3). These results highlight the importance of the protonated form for intercalation into the interlayer space of the Na-MMT by the cation exchange process, since drugs with these properties were able to intercalate Na-NMT (Table 2). All of the predominant species of these drugs demonstrated one favorable protonation site except for CLX, which presented four favorable protonation points (Fig. 2).

**Fig 3:**
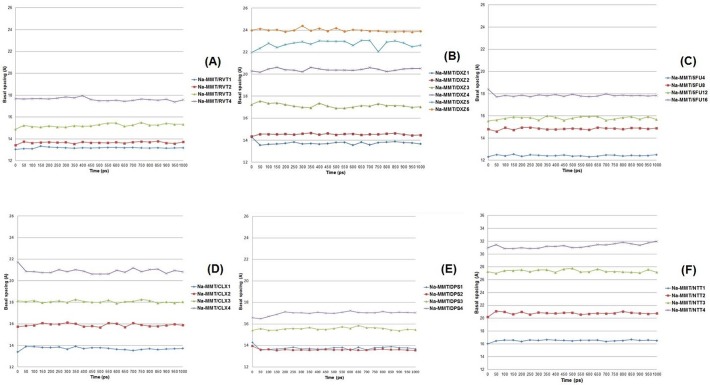
The variation in basal spacing distance in the Na-MMT/amine-containing drugs models during 1 ns molecular dynamics simulations.

Herein we built different models of the molecular systems in which the amine-containing drugs intercalated with Na-MMT. Each model contained different numbers of drug molecules to find the molecular system model that had similar basal spacing compared to the experimental results in order to study the molecular arrangement. The Na-MMT model had an *a* axis (15.6 Å) and *b* axis (18.4 Å) with four Na^+^ atoms that were substituted by protonated amine-drug molecules when it was necessary to maintain neutrality of the molecular systems models. Therefore, the models containing non-ionized 5FU were built with four Na^+^ atoms present in the interlayer spacing. The variations in basal spacing are shown on 1000 ps (1 ns) of molecular dynamics simulation in Fig. 3.

The drug RVT is a cholinergic (butyrylcholinesterase and acetylcholinesterase) inhibitor used for the treatment of the Alzheimer’s disease [39]. In this work, protonated RVT (predominant microspecie) was used to build Na-MMT/RVT models to study the interaction and arrangement of the molecular drug in the interlayer space. Using molecular dynamics simulations, four different models (Na-MMT/RVT1, Na-MMT/RVT2, Na-MMT/RVT3 and Na-MMT/RVT4) were built and optimized to observe whether differences occur in the basal spacing distance due to incorporation of different numbers of RVT molecules.

Analysis of Na-MMT/RVT1 and Na-MMT/RVT2 by molecular dynamics simulation indicated that these complexes had reduced basal spacing, smaller than 14 Å (Fig. 3). In addition, the RVT molecules were found to have a planar arrangement in relation to the lamellar surface. In contrast, Na-MMT/RVT3 was found to have varied basal spacing values ranging from 14.8 Å to 15.7 Å, where three RVT molecules were affixed to the surface of MMT, resulting in slight displacement of the basal spacing compared with both previous models.

The simulation studies of Na-MMT/RVT4 model pointed to the basal spacing values ranged between 17.3 Å to 18.0 Å as the closest to the experimental result (17.9 Å, shown in Table 2). The presence of four RVT molecules in Na-NMT caused the amine groups to point toward the lamellar surface. The protonated amine group of two RVT molecules was positioned toward Layer 1 while the other two molecules pointed the protonated amine group toward Layer 2. This result suggests that these types of arrangements are able to increase basal spacing to 17.9 Å. Additionally, it was found that RVT adopted a molecular orientation in the z-direction and formed a vertical monolayer arrangement. The basal spacing average of the Na-MMT/RVT4 model was 17.62 Å (Table 2).

The drug DXZ is a quinazoline derivative and α-1-adrenergic receptor inhibitor used to treat high blood pressure and urinary retention associated with benign prostatic hyperplasia [40]. Herein, we built six Na-MMT/DXZ models (Na-MMT/DXZ1, Na-MMT/DXZ2, Na-MMT/DXZ3, Na-MMT/DXZ4, Na-MMT/DXZ5, Na-MMT/DXZ6) to observe how the arrangement of protonated DXZ molecules influenced basal spacing. Results obtained from the Na-MMT/DXZ1, Na-MMT/DXZ2 and Na-MMT/DXZ3 complexes indicate that the basal spacing was lower than 18 Å (Fig. 3) and that the protonated DXZ molecules lie upon the surface of MMT. However, Na-MMT/DXZ4, Na-MMT/DXZ5 and Na-MMT/DXZ6 models had basal spacing values greater than 20 Å. When assessing the basal spacing of Na-MMT/DXZ6, which incorporated six protonated DXZ into the interlayer space, the value was found to be very close to the experimental results obtained (24.2 Å), shown in Table 2.

The molecular dynamics simulation of Na-MMT/DXZ6 indicated that the basal spacing varies between 23.8 Å to 24.3 Å. The snapshot of the protonated DXZ arrangement and the basal spacing of Na-MMT/DXZ6 at 1 ns was 23.90 Å. Our molecular modeling data showed that protonated DXZ molecules nearest to the Na-MMT lamellar surface are arranged so as to direct the amine groups of the quinazoline moiety to the sheet surface. The basal spacing average of the Na-MMT/DXZ6 model was 23.98 Å (Table 2).

The drug 5FU is a pyrimidine analog that inhibits the biosynthesis of deoxyribonucleotides for DNA replication, targeting thymidylate synthase that causes thymidine depletion and cell death. It also has application as an antineoplasic drug [41]. Markova et al. (2010) studied 5FU under different pH conditions showing that its neutral population was higher than the ionized populations at a neutral pH [42]. The intercalation reaction was conducted in aqueous media (pH ~5.0), therefore according to our findings the neutral form of 5FU was the predominant population (Table 3). Since 5FU had the smallest volume (93.61 Å^3^) of all the amine-containing drugs included in this study (Table 3), we prepared four Na-MMT/5FU complex models (Na-MMT/5FU4, Na-MMT/5FU8, Na-MMT/5FU12, Na-MMT/5FU16), each with a different number of molecules. Lin et al. (2002) successfully performed the intercalation of 5FU with the Na-MMT interlayer while testing different conditions, in which the total amount of 5FU that successfully intercalated was 87.5 mg for each gram of Na-MMT [43], close to our result of 89.2 mg shown in Table 2. Our molecular dynamics study showed that the intercalation mechanism of 5FU under 298 K was likely due to adsorption to the free surface as well as formation of hydrogen bonds between the amine group hydrogen on the pyrimidine ring and lamellar surface oxygen atoms of Na-MMT.

The experimental basal spacing found for Na-MMT/5FU was 15.8 Å, shown in Table 2. The basal spacing found for Na-MMT/5FU4 was smaller than 14 Å. This value was based upon the model where all 5FU neutral molecules were adsorbed in parallel to the lamellar surface without formation of hydrogen bonds between the drug molecules and the Na-MMT surface. The Na-MMT/5FU8 and Na-MMT/5FU12 models showed increased basal spacing distance of Na-MMT to which the drug molecules are adsorbed. This absorption occurs by interaction of the pyrimidine ring amine group at the Na-MMT surface and formation of hydrogen bonds with the oxygen of the sheet surface. The Na-MMT/5FU12 model showed a very similar basal spacing distance to our experimental result, ranging from 15.4 Å to 16.1 Å in 1 ns molecular dynamics simulation as can be seen in Fig. 3. The basal spacing distance of the Na-MMT/5FU16 complex varied near 18 Å with hydrogen bonding between 5FU and oxygen atoms at the layer surface. The basal spacing average of the Na-MMT/5FU12 model was 15.79 Å (Table 2).

CLX is a cationic biguanidine drug that is used as an antimicrobial agent. Meng et al. (2009) reported that CLX intercalates with Na-MMT and that its antimicrobial activity is directed against inhibiting the growth of diverse microorganisms such as Gram-positive bacteria and Gram-negative bacteria [44]. In our study we conducted the intercalation reaction in aqueous media at pH ~5.0, where protonated CLX was the predominant form in the population (Table 3). We performed theoretical constructions of four Na-MMT/CLX models (Na-MMT/CLX1, Na-MMT/CLX2, Na-MMT/CLX3, Na-MMT/CLX4) using increasing numbers of protonated CLX molecules. The experimental basal spacing of Na-MMT/CLX is 17.5 Å as shown in Table 2.

The Na-MMT/CLX1 model had a basal spacing distance smaller than 14 Å, as shown in Fig. 3, while the CLX molecule adopts a planar adsorbed arrangement on the surface of Na-MMT. Na-MMT/CLX2 model had a basal spacing value between 15.6 Å to 16.1 Å and the CLX molecules adopted a lateral monolayer arrangement. The Na-MMT/CLX3 model had a basal spacing value that ranged between 17.8 Å to 18.3 Å and the CLX molecules adopted a monolayer arrangement. Each guanidine group of one CLX molecule interacted with a different surface of MMT.

Our data showed that the Na-MMT/CLX3 model had a basal spacing value closer to the experimental result (17.5 Å) than any other constructed models. CLX is able to perform hydrogen bonding interactions with oxygen atoms at the lamellar surface as well with ion-dipole interactions that occur due to protonation of the amine groups. The Na-MMT/CLX4 model had its basal spacing value varied between 20.6 Å to 21.7 Å with guanidine groups interacting with Na-MMT surface. The basal spacing average of the Na-MMT/CLX3 model was 18.07 Å (Table 2).

The compound DPS is a drug that is used to treat infectious diseases including leprosy, tuberculosis, malaria and AIDS-related pneumonia [45]. DPS was intercalated in a media containing 1N HCl at pH ~2.0. At this pH the predominant forms of DPS was protonated (Table 3) and the experimental basal spacing value obtained for Na-MMT/DPS was 15.7 Å (Table 2). In this study we prepared four Na-MMT/DPS models (Na-MMT/DPS1, Na-MMT/DPS2, Na-MMT/DPS3, Na-MMT/DPS4) using differing numbers of protonated DPS molecules.

The Na-MMT/DPS1 and Na-MMT/DPS2 models had a basal spacing distance close to 14 Å, and DPS adopted a planar adsorbed arrangement at the Na-MMT surface. Na-MMT/DPS3 model showed a basal spacing value between 15.41 Å to 15.86 Å (Fig. 3), in which the DPS molecules adopted an inclined monolayer arrangement with a protonated amine group oriented to the lamellar planes whereas the molecular dynamics simulations showed the basal spacing value near to 15.7 Å at 1 ns. This basal spacing value was also very close to the experimental result (Table 2). Na-MMT/DPS4 model had a basal spacing value that ranged between 16.49 Å to 17.23 Å. The molecular arrangement of DPS in Na-MMT/DPS4 model follows the same orientation as Na-MMT/DPS3. Thus these findings indicate that this molecular arrangement for DPS can occur in the interlayer gallery. The basal spacing average of the Na-MMT/DPS3 model was 15.55 Å (Table 2).

NTT is a polyene antifungal drug that is used to treat systemic fungal infections [46]. In this study we performed the intercalation experiment in media containing acetic acid 0.9% at pH ~2.8, where protonated NTT exists as the predominant form, shown in Table 3. The experimental basal spacing obtained for Na-MMT/NTT was 16.9 Å (Table 2). Furthermore, Pupe and coworkers (2011) also successfully performed inclusion of NTT into Na-MMT and showed the potential of this complex as a delivery drug system [32]. Therefore, in order to study the interaction of NTT with Na-NMT, four Na-MMT/NTT models (Na-MMT/NTT1, Na-MMT/NTT2, Na-MMT/NTT3, Na-MMT/NTT4) were prepared using a different number of protonated NTT molecules as shown in Fig. 2.

**Fig 4:**
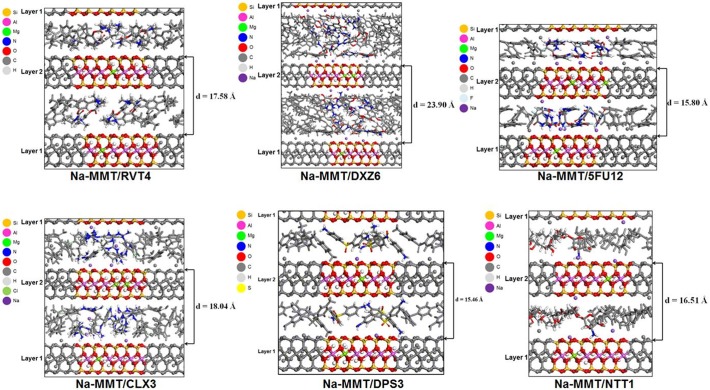
Snapshots at 1ns of the molecular dynamics simulation of the Na-MMT/drugs models showing the basal spacing. Structures in gray represent the periodic boundary conditions. The projection view is shown along the *b*-axis.

Na-MMT/NTT1 model was found to have a basal spacing that ranged between 16.0–16.6 Å, so this finding was closer to the experimentally obtained result than the other models studied, shown in Fig. 3. The NTT molecule adopted a planar monolayer arrangement in the interlayer gallery of Na-MMT. The protonated amine group of NTT was shown to occupy a central location in the octahedral cavity of the Na-MMT sheet along with the Na^+^ cations. The Na-MMT/NTT2 model was found to have a basal spacing value ranging between 20.1 Å to 21.1 Å, and the NTT molecules were arranged in a lateral monolayer and the protonated amine groups remained distant from the lamellae. Furthermore, the Na-MMT/NTT3 model had a basal spacing value in the 27.0–27.7 Å range, and the NTT molecules adopted a lateral bilayer arrangement. The Na-MMT/NTT4 model had a basal spacing distance that ranged 30.8–31.9 Å and the NTT molecules adopted an inclined arrangement. The basal spacing average of the Na-MMT/NTT1 model was 16.51 Å (Table 2). The molecular arrangements of the amine-containing drugs and basal spacing are showed in Fig. 4.

### Interaction Energies, and Interrelations between Drugs Molecular Properties and Experimental Observations

Interaction energy and the molecular arrangement are important properties which determine the ability of a drug to absorb to the lamellar surface of Na-MMT, therefore molecular dynamics (MD) simulations were performed to determine these values and properties. The main microspecie found for RVT, DXZ, 5FU, CLX, DPS and NTT was used to build models of different Na-MMT/amine-containing drugs. The interaction energies of Na-MMT/amine-containing drugs complexes were obtained by MD simulations. The energy component terms considered here were bonded and non-bonded interactions including (van der Waals and electrostatic terms involved in interaction energy).

The Na-MMT/RVT4, Na-MMT/DXZ6, Na-MMT/5FU12, Na-MMT/CLX3, Na-MMT/DPS3 and Na-MMT/NTT1 models displayed similar basal spacing compared to what was determined experimentally by XRPD. These similar results may suggest the kind of the molecular arrangement of these drugs occurred within the interlayer space. Herein we examined the interaction energies of intercalated drugs molecules with the lamellar surface in order to identify the amine-containing drug possessing the most favorable orientation toward Na-MMT. Interaction energy is an important parameter to consider when assessing differences of the energies between drug compounds. Thus we calculated the average interaction energies between the drugs and both layers of the models in the dry state corresponding to the equilibrated system trajectory at *T* = 298 K (Table 4).

**Table 4:** Average interaction energies (kcal/mol) of the amine-containing drugs with the lamellar surface in the interlayer space of the Na-MMT.

Drug	Average interaction energy (kcal/mol)*
RVT	−10.23
DXZ	12.88
5FU	−3.64
CLX	−19.87
DPS	−22.81
NTT	−42.36

*The averages interaction energies with lamellar surfaces in the interlayer spacing were obtained by considering the interaction energies with both lamellar surfaces.

The interaction energy was calculated for the molecules in the models with basal spacing similar to the experimental results. In the Na-MMT/RVT4 model, RVT adopted a vertical monolayer arrangement (S1 Fig.). However the direction of the protonated amine group alternated pointing to different surfaces in MMT. RVT molecules showed the average interaction energy (-10.23 kcal/mol) more favorable (stronger interaction) with MMT than DXZ and 5FU (Table 4). In Na-MMT/DXZ6 model, the DXZ molecules adopted an overlapping arrangement, which created a bilayer with the amine groups of the quinazoline moiety of DXZ molecules closest to the Na-MMT layer oriented toward the sheet surface. DXZ molecules were found to have the weaker interaction of all intercalated drugs with MMT (Table 4).

In the Na-MMT/5FU12 model, a couple of the 5FU molecules adopted a mixture of standing on end and lying-flat arrangements, thus creating an 5FU network maintained by hydrogen bonds and the NH group was directed toward the surface layer. 5FU molecules showed the average interaction energy more favorable with MMT than DXZ molecules. The approach of the 5FU carbonyl groups to the oxide surface of Na-MMT may have hindered the interaction of this moiety of 5FU molecules with the layer.

In the Na-MMT/CLX3 model the CLX molecules adopted a monolayer arrangement in which each guanidine group of the molecules were directed towards different surfaces of MMT. CLX molecules showed stronger interaction energy with MMT than DXZ, 5FU and RVT. In the Na-MMT/DPS3 model, DPS adopted an inclined monolayer arrangement in which amine group was oriented to the lamellar planes. DPS molecules showed the interaction energy more favorable with MMT than CLX. In the Na-MMT/NTT1 model, the polyene moiety of the NTT molecule adopted a slightly planar monolayer arrangement. NTT1 molecule showed favorable interaction energies with MMT where the protonated amine group toward in the octahedral cavity of the lamellar surface. The DPS and NTT molecules had lower interaction energies when intercalated with the Na-MMT than other amine-containing drugs. This may be related to the position of the amine group at the Na-MMT octahedral cavity. In addition, DPS and NTT were found to have the smallest contact distance from the Na-MMT lamellar surface.

The distance on 1 ns of drugs molecules from MMT lamellar surface viewed from de *c* axis is showed in S2 Fig. In Na-MMT/RVT4 model the methyl groups bonded with protonated amine group in RVT molecules lodged on octahedral cavity area performing interaction with the oxygen atoms of MMT lamellar surface. Unlike the organized arrangement observed for the RVT molecules in the interlayer space of the Na-MMT/RVT4 model, DXZ molecules showed no pattern conformational arrangement in the interlayer space of Na-MMT/DXZ6 model. It is likely that not all DXZ molecules interacted with the lamellar octahedral cavity of the MMT by their protonated amine group. The non-ionized 5FU molecules of Na-MMT/5FU12 model interact with the oxygen atoms of the lamellar surface. The protonated CLX molecules of the Na-MMT/CLX3 model interact with the oxygen atoms of the lamellar surface by amine group of the guanidine moiety. The protonated DPS molecules of the Na-MMT/DPS3 model lodged the amine group protonated on the octahedral cavity area performing interaction with the oxygen atoms of MMT lamellar surface, likewise the protonated NTT molecule in the Na-MMT/NTT1 model. DPS and NTT molecules showed the lower interaction energy than the other amine-containing drugs, presenting the adsorption on a central area of the octahedral cavity.

The ascending order of the interaction strength of the amine-containing drugs in interlayer spacing of the Na-MMT is DXZ<5FU<RVT<CLX<DPS<NTT. These results showed no direct interrelationship between interaction strength and basal spacing distance. However, the interaction strength may influence the drugs molecules diffusion, and the drugs molecules with smaller strength interaction may diffuse more easily both in and out from the interlayer gallery, while drugs molecules with stronger interaction may adsorb strongly on lamellar surface and so diffuse more slowly. Our molecular dynamics simulations were performed in the dry state, and the presence of water in the experimental intercalation could somewhat change the basal plane spacing reported (swelling properties and hysteresis), so simulations in aqueous environment shall be conducted in the future to understand the influence of water on the strength of the drugs molecules adsorption in the interlayer gallery and basal spacing plane distance.

**Fig 5:**
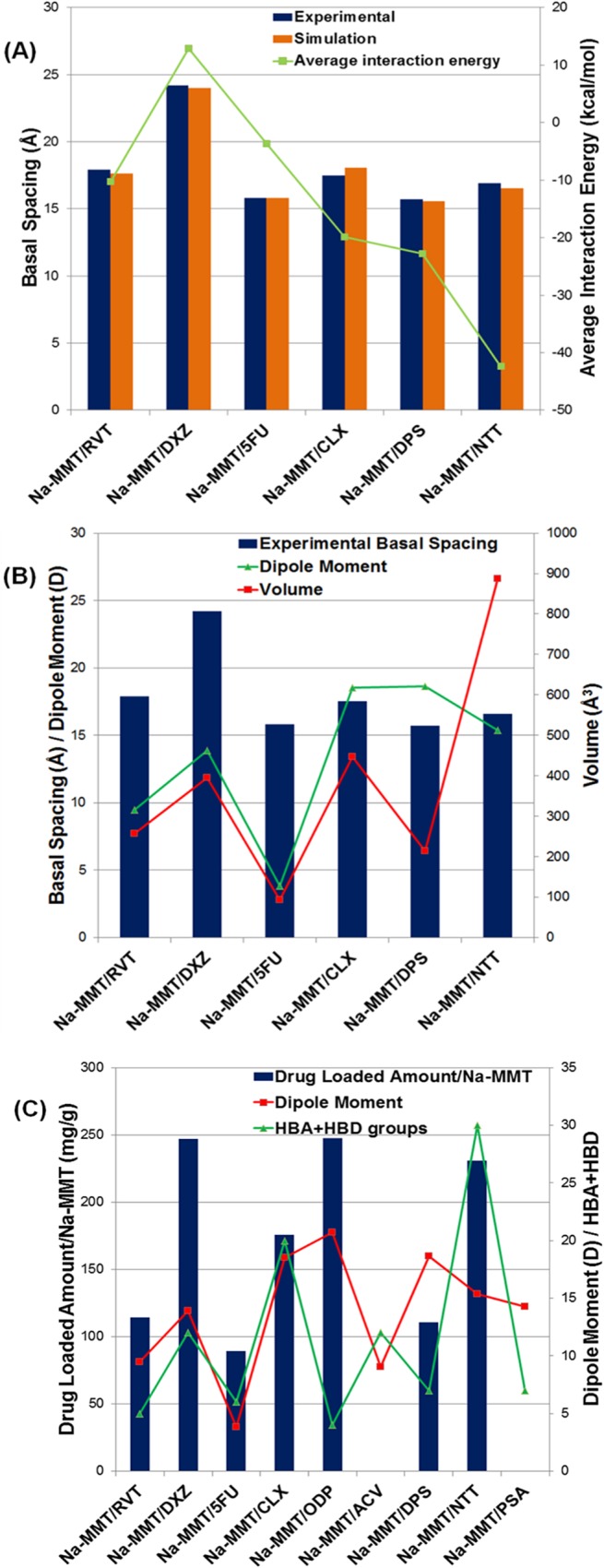
Amine-containing drugs molecular properties and experimental observations. (A) Comparison of the experimental and simulate basal spacing, and average interaction energies. (B) Interrelations between experimental basal spacing and dipole moment and molecular volume. (C) Interrelations between drug-loaded amount/Na-MMT and dipole moment and number of the HBA+HBD groups.

Moment dipole and volume are molecular properties of the amine-containing drugs which may have interrelation with the displacement of basal spacing plane of the Na-MMT (Fig. 5). Our results suggest that these molecular properties play an important role in increasing the basal spacing, but this displacement can be affected by other factors. Likewise, moment dipole and both HBA and HBD groups number may be important in the drug-loaded amount in the Na-MMT, but also other factors as pH and lipophilicity of the drugs can affect this experimental observation.

## Conclusion

All experimental results were confirmed using the FT-IR and DSC techniques and characterized by XRPD analysis. Experimental assays indicated that ODP, DXZ and NTT had the highest agent-loaded values, and of all the compounds tested, DXZ was found to have the largest interlayer spacing, but ODP showed low interlayer space indicating no efficient intercalation. Two drug compounds ACV and PSA, however were found to be no intercalated candidates in experimental conditions used, since we were unable to find evidence that they intercalated the Na-MMT complexes.

The approach using the molecular modeling tools allowed to find the concentrations of nonprotonated and protonated drugs according to the experimental conditions used herein. This study revealed new information and indicated that intercalated protonated amine groups of RVT, DXZ, CLX, DPS and NTT were likely involved in ion-dipole and hydrogen bonding interactions with Na-MMT lamellar surface. The drug compound PSA showed predominately in the deprotonated form indicating that it does not have a favorable form for intercalation with Na-MMT. The drug ACV also exists in protonated forms with no evidence of intercalation, probably due to the influence of acidic conditions present in the reaction experiment. 5FU showed a prevalent concentration of neutral drug forms and evidence of intercalation, suggesting that this intercalation process is likely due to free adsorption and hydrogen bonding interactions.

The arrangement of different amine-containing drugs and their orientation with Na-MMT are important insight on the interlayer aspect of the molecular system formed to design improved drug delivery systems based on Na-MMT.

## Supporting Information

S1 Fig Snapshots at 1ns of molecular dynamics simulation of the Na-MMT/drugs models showing the molecular arrangement of the drugs along the Na-MMT interlayer space. The projection view is shown along the *b*-axis.
(TIF)

S2 Fig Snapshots at 1ns of molecular dynamics simulation of the Na-MMT/drugs models showing the distance from and the interactions of the drugs molecules with the lamellar surface of MMT. The projection view is shown along the *c*-axis.
(TIF)

S1 Table Basal spacing (Å) of Na-MMT/RVT models every 50 ps along 1ns of molecular dynamics simulation. (DOCX)

S2 Table Basal spacing (Å) of Na-MMT/DXZ models every 50 ps along 1ns of molecular dynamics simulation. (DOCX)

S3 Table Basal spacing (Å) of Na-MMT/5FU models every 50 ps along 1ns of molecular dynamics simulation. (DOCX)

S4 Table Basal spacing (Å) of Na-MMT/CLX models every 50 ps along 1ns of molecular dynamics simulation. (DOCX)

S5 Table Basal spacing (Å) of Na-MMT/DPS models every 50 ps along 1ns of molecular dynamics simulation. (DOCX)

S6 Table Basal spacing (Å) of Na-MMT/NTT models every 50 ps along 1ns of molecular dynamics simulation. (DOCX)
