# Supplementary figures and images for: Sodium Montmorillonite/Amine-Containing Drugs Complexes: New Insights on Intercalated Drugs Arrangement into Layered Carrier Material
Source: PLoS One. 2015 Mar 24;10(3):e0121110. doi: 10.1371/journal.pone.0121110 (PMC4372448; doi:10.1371/journal.pone.0121110)

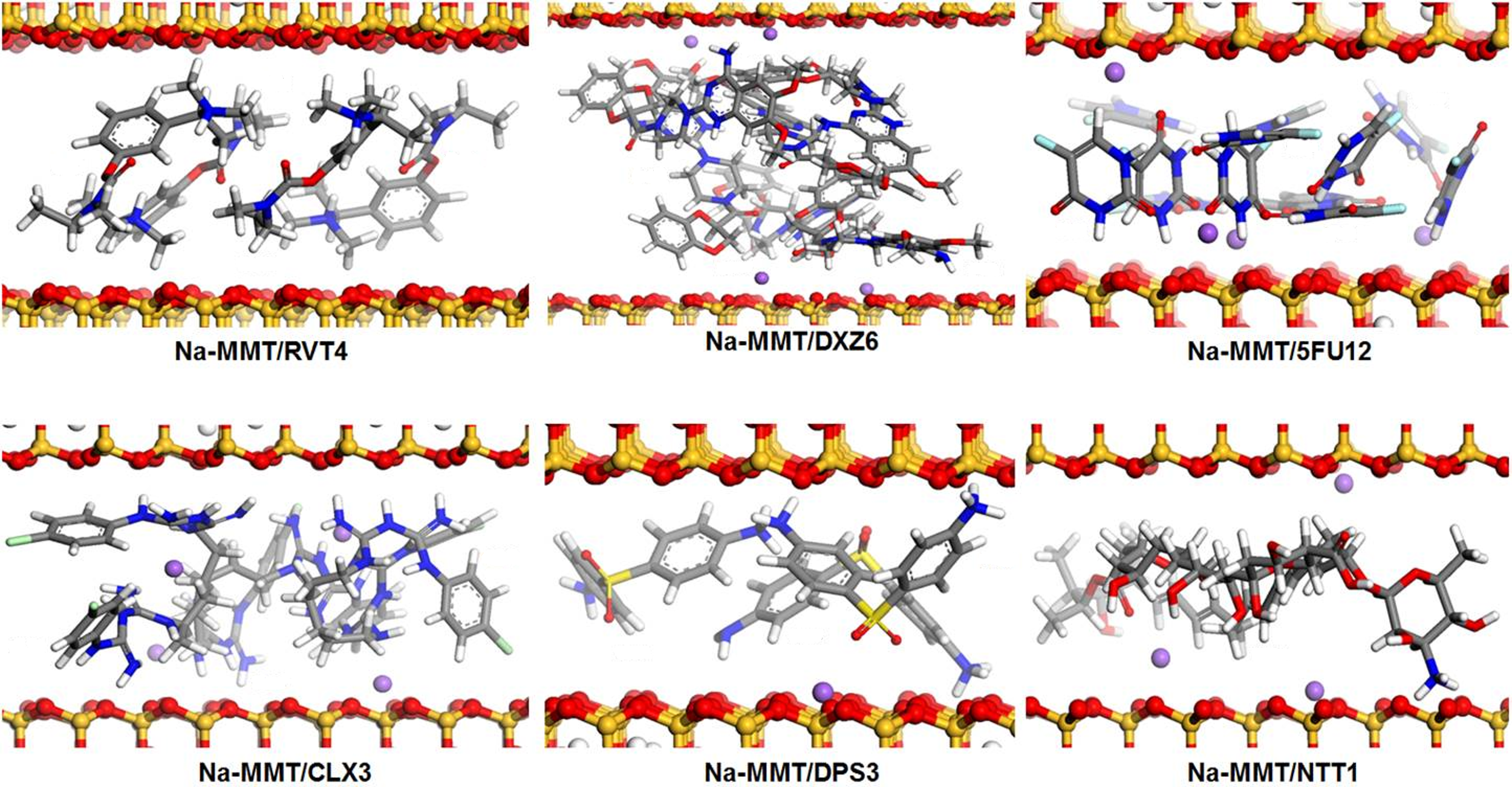

Supplement: S1 Fig — The projection view is shown along the b-axis. (TIF) [file pone.0121110.s001.tif]

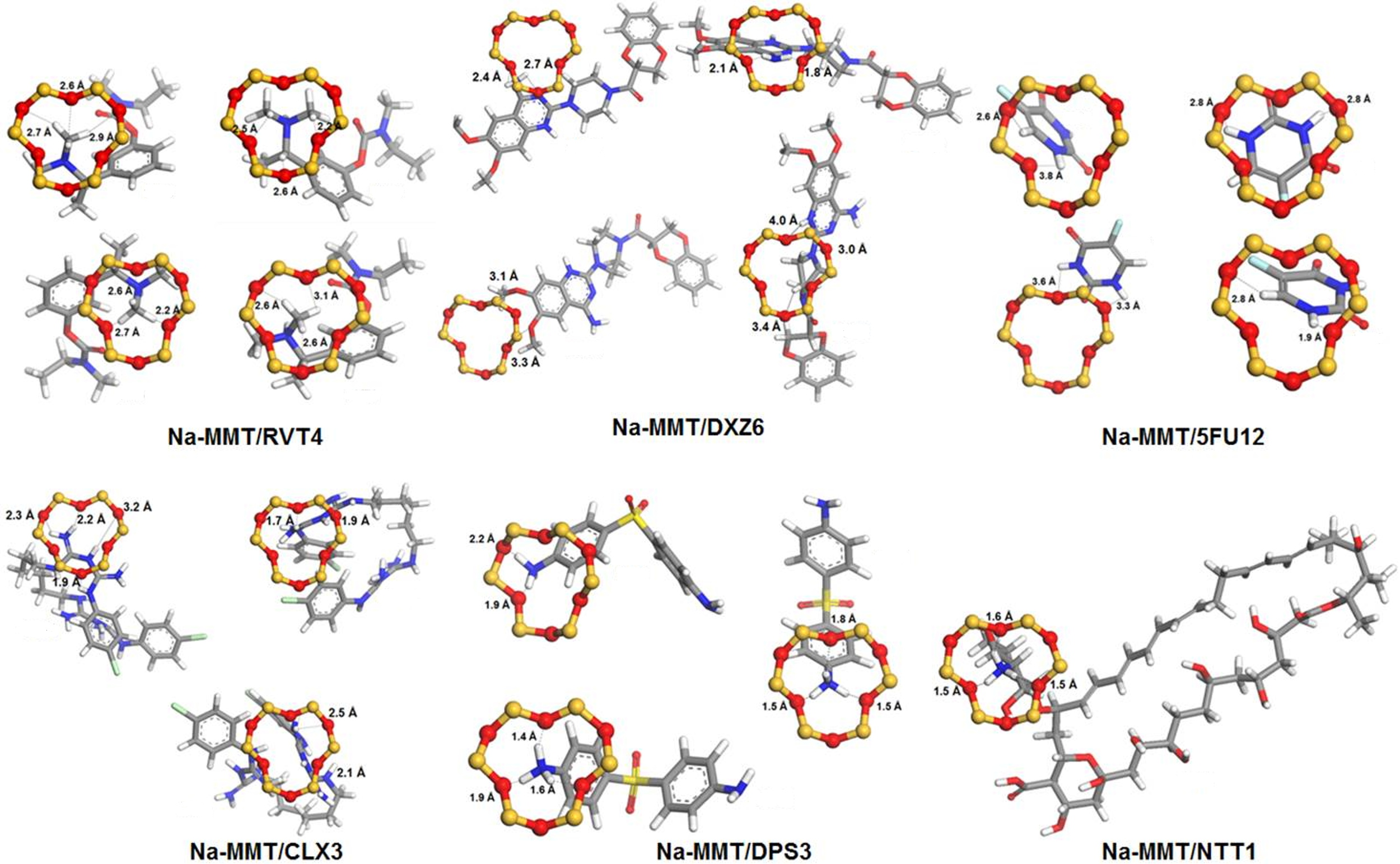

Supplement: S2 Fig — The projection view is shown along the c-axis. (TIF) [file pone.0121110.s002.tif]
