# Supplementary material for: Sodium Montmorillonite/Amine-Containing Drugs Complexes: New Insights on Intercalated Drugs Arrangement into Layered Carrier Material
Source: PLoS One. 2015 Mar 24;10(3):e0121110. doi: 10.1371/journal.pone.0121110 (PMC4372448; doi:10.1371/journal.pone.0121110)
Supplement: S1 Table — (DOCX) [file pone.0121110.s003.docx]

**Table S1. Basal spacing (Å) of Na-MMT/RVT models every 50 ps along 1ns of molecular dynamics simulation.**

| **Time** | **Basal spacing (Å)** | | | |  |
| --- | --- | --- | --- | --- | --- |
|  | **Na-MMT/RVT1** | **Na-MMT/RVT2** | **Na-MMT/RVT3** | **Na-MMT/RVT4** |  |
| 0 | 13.03 | 13.42 | 14.89 | 17.67 |  |
| 50 | 13.11 | 13.74 | 15.22 | 17.64 |  |
| 100 | 13.09 | 13.62 | 15.12 | 17.67 |  |
| 150 | 13.34 | 13.67 | 15.07 | 17.68 |  |
| 200 | 13.25 | 13.70 | 15.18 | 17.66 |  |
| 250 | 13.22 | 13.66 | 15.09 | 17.72 |  |
| 300 | 13.18 | 13.69 | 15.08 | 17.83 |  |
| 350 | 13.15 | 13.54 | 15.20 | 17.76 |  |
| 400 | 13.18 | 13.71 | 15.16 | 17.95 |  |
| 450 | 13.16 | 13.65 | 15.18 | 17.61 |  |
| 500 | 13.18 | 13.65 | 15.31 | 17.48 |  |
| 550 | 13.22 | 13.63 | 15.44 | 17.48 |  |
| 600 | 13.21 | 13.69 | 15.46 | 17.54 |  |
| 650 | 13.18 | 13.59 | 15.16 | 17.44 |  |
| 700 | 13.22 | 13.70 | 15.27 | 17.52 |  |
| 750 | 13.17 | 13.76 | 15.50 | 17.65 |  |
| 800 | 13.16 | 13.70 | 15.25 | 17.59 |  |
| 850 | 13.18 | 13.78 | 15.26 | 17.53 |  |
| 900 | 13.15 | 13.62 | 15.43 | 17.63 |  |
| 950 | 13.17 | 13.57 | 15.33 | 17.38 |  |
| 1000 | 13.18 | 13.71 | 15.34 | 17.58 |  |
| Average | 13.17 | 13.65 | 15.23 | 17.62 |  |
