# Supplementary material for: Sodium Montmorillonite/Amine-Containing Drugs Complexes: New Insights on Intercalated Drugs Arrangement into Layered Carrier Material
Source: PLoS One. 2015 Mar 24;10(3):e0121110. doi: 10.1371/journal.pone.0121110 (PMC4372448; doi:10.1371/journal.pone.0121110)
Supplement: S2 Table — (DOCX) [file pone.0121110.s004.docx]

**Table S2. Basal spacing (Å) of Na-MMT/DXZ models every 50 ps along 1ns of molecular dynamics simulation.**

| **Time** | **Basal spacing (Å)** | | | | | |  |
| --- | --- | --- | --- | --- | --- | --- | --- |
|  | **Na-MMT/DXZ1** | **Na-MMT/DXZ2** | **Na-MMT/DXZ3** | **Na-MMT/DXZ4** | **Na-MMT/DXZ5** | **Na-MMT/DXZ6** |  |
| 0 | 14.28 | 14.35 | 17.26 | 20.28 | 21.98 | 24.00 |  |
| 50 | 13.56 | 14.56 | 17.55 | 20.17 | 22.36 | 24.13 |  |
| 100 | 13.64 | 14.56 | 17.35 | 20.48 | 22.81 | 24.00 |  |
| 150 | 13.67 | 14.54 | 17.39 | 20.62 | 22.42 | 24.03 |  |
| 200 | 13.72 | 14.57 | 17.24 | 20.40 | 22.69 | 23.84 |  |
| 250 | 13.84 | 14.52 | 17.10 | 20.36 | 22.83 | 23.98 |  |
| 300 | 13.67 | 14.59 | 17.00 | 20.22 | 22.95 | 24.39 |  |
| 350 | 13.71 | 14.66 | 16.96 | 20.61 | 22.72 | 23.96 |  |
| 400 | 13.66 | 14.52 | 17.36 | 20.49 | 23.02 | 24.16 |  |
| 450 | 13.69 | 14.62 | 17.10 | 20.38 | 23.00 | 23.91 |  |
| 500 | 13.80 | 14.50 | 16.91 | 20.36 | 22.99 | 24.19 |  |
| 550 | 13.82 | 14.57 | 16.92 | 20.36 | 22.99 | 23.87 |  |
| 600 | 13.56 | 14.58 | 17.01 | 20.35 | 22.62 | 24.03 |  |
| 650 | 13.83 | 14.49 | 17.15 | 20.44 | 23.07 | 23.99 |  |
| 700 | 13.58 | 14.54 | 17.11 | 20.60 | 23.05 | 23.91 |  |
| 750 | 13.79 | 14.54 | 17.31 | 20.45 | 22.05 | 23.93 |  |
| 800 | 13.83 | 14.60 | 17.15 | 20.24 | 22.92 | 23.84 |  |
| 850 | 13.89 | 14.64 | 17.14 | 20.35 | 23.03 | 23.84 |  |
| 900 | 13.79 | 14.53 | 17.13 | 20.47 | 22.83 | 23.87 |  |
| 950 | 13.77 | 14.45 | 16.99 | 20.52 | 22.49 | 23.83 |  |
| 1000 | 13.67 | 14.47 | 17.04 | 20.52 | 22.62 | 23.90 |  |
| Average | 13.75 | 14.54 | 17.15 | 20.41 | 22.73 | 23.98 |  |
