# Supplementary material for: Sodium Montmorillonite/Amine-Containing Drugs Complexes: New Insights on Intercalated Drugs Arrangement into Layered Carrier Material
Source: PLoS One. 2015 Mar 24;10(3):e0121110. doi: 10.1371/journal.pone.0121110 (PMC4372448; doi:10.1371/journal.pone.0121110)
Supplement: S3 Table — (DOCX) [file pone.0121110.s005.docx]

**Table S3. Basal spacing (Å) of Na-MMT/5FU models every 50 ps along 1ns of molecular dynamics simulation.**

| **Time** | **Basal spacing (Å)** | | | |  |
| --- | --- | --- | --- | --- | --- |
|  | **Na-MMT/5FU4** | **Na-MMT/5FU8** | **Na-MMT/5FU12** | **Na-MMT/5FU16** |  |
| 0 | 12.31 | 14.81 | 15.55 | 18.40 |  |
| 50 | 12.50 | 14.60 | 15.64 | 17.71 |  |
| 100 | 12.40 | 14.94 | 15.77 | 17.83 |  |
| 150 | 12.55 | 14.73 | 15.91 | 17.75 |  |
| 200 | 12.36 | 14.96 | 15.85 | 17.86 |  |
| 250 | 12.49 | 14.95 | 15.85 | 17.72 |  |
| 300 | 12.45 | 14.87 | 15.62 | 17.89 |  |
| 350 | 12.40 | 14.79 | 16.01 | 17.81 |  |
| 400 | 12.41 | 14.79 | 15.88 | 17.92 |  |
| 450 | 12.48 | 14.83 | 15.61 | 17.77 |  |
| 500 | 12.37 | 14.87 | 15.79 | 17.95 |  |
| 550 | 12.40 | 14.83 | 15.95 | 17.78 |  |
| 600 | 12.31 | 14.75 | 15.94 | 17.74 |  |
| 650 | 12.37 | 14.95 | 15.96 | 17.80 |  |
| 700 | 12.48 | 14.89 | 15.60 | 17.98 |  |
| 750 | 12.47 | 14.87 | 15.72 | 17.81 |  |
| 800 | 12.39 | 14.80 | 15.85 | 17.86 |  |
| 850 | 12.44 | 14.91 | 15.93 | 17.82 |  |
| 900 | 12.41 | 14.90 | 15.70 | 17.84 |  |
| 950 | 12.42 | 14.83 | 15.93 | 17.79 |  |
| 1000 | 12.50 | 14.90 | 15.68 | 17.83 |  |
| Average | 12.42 | 14.84 | 15.79 | 17.85 |  |
