# Supplementary material for: Sodium Montmorillonite/Amine-Containing Drugs Complexes: New Insights on Intercalated Drugs Arrangement into Layered Carrier Material
Source: PLoS One. 2015 Mar 24;10(3):e0121110. doi: 10.1371/journal.pone.0121110 (PMC4372448; doi:10.1371/journal.pone.0121110)
Supplement: S4 Table — (DOCX) [file pone.0121110.s006.docx]

**Table S4. Basal spacing (Å) of Na-MMT/CLX models every 50 ps along 1ns of molecular dynamics simulation.**

| **Time** | **Basal spacing (Å)** | | | |  |
| --- | --- | --- | --- | --- | --- |
|  | **Na-MMT/CLX1** | **Na-MMT/CLX2** | **Na-MMT/CLX3** | **Na-MMT/CLX4** |  |
| 0 | 13.40 | 15.74 | 18.13 | 21.73 |  |
| 50 | 13.90 | 15.83 | 18.08 | 20.87 |  |
| 100 | 13.89 | 15.87 | 18.15 | 20.84 |  |
| 150 | 13.82 | 16.08 | 17.95 | 20.75 |  |
| 200 | 13.81 | 15.98 | 18.05 | 20.75 |  |
| 250 | 13.86 | 15.97 | 18.14 | 21.02 |  |
| 300 | 13.66 | 16.12 | 17.99 | 20.84 |  |
| 350 | 13.92 | 16.02 | 18.26 | 21.02 |  |
| 400 | 13.71 | 15.75 | 18.08 | 20.88 |  |
| 450 | 13.79 | 15.79 | 18.00 | 20.61 |  |
| 500 | 13.79 | 15.68 | 18.01 | 20.61 |  |
| 550 | 13.73 | 16.08 | 18.20 | 20.62 |  |
| 600 | 13.64 | 16.03 | 17.90 | 20.96 |  |
| 650 | 13.62 | 15.70 | 18.06 | 20.79 |  |
| 700 | 13.53 | 16.07 | 18.09 | 21.19 |  |
| 750 | 13.63 | 15.90 | 18.27 | 20.85 |  |
| 800 | 13.69 | 15.78 | 18.16 | 21.03 |  |
| 850 | 13.61 | 15.78 | 17.92 | 21.08 |  |
| 900 | 13.67 | 15.87 | 18.04 | 20.67 |  |
| 950 | 13.69 | 15.96 | 17.94 | 20.95 |  |
| 1000 | 13.72 | 15.88 | 18.04 | 20.83 |  |
| Average | 13.71 | 15.89 | 18.07 | 20.90 |  |
