# Supplementary material for: Sodium Montmorillonite/Amine-Containing Drugs Complexes: New Insights on Intercalated Drugs Arrangement into Layered Carrier Material
Source: PLoS One. 2015 Mar 24;10(3):e0121110. doi: 10.1371/journal.pone.0121110 (PMC4372448; doi:10.1371/journal.pone.0121110)
Supplement: S5 Table — (DOCX) [file pone.0121110.s007.docx]

**Table S5. Basal spacing (Å) of Na-MMT/DPS models every 50 ps along 1ns of molecular dynamics simulation.**

| **Time** | **Basal spacing (Å)** | | | |  |
| --- | --- | --- | --- | --- | --- |
|  | **Na-MMT/DPS1** | **Na-MMT/DPS2** | **Na-MMT/DPS3** | **Na-MMT/DPS4** |  |
| 0 | 14.28 | 13.94 | 15.41 | 16.59 |  |
| 50 | 13.56 | 13.60 | 15.57 | 16.49 |  |
| 100 | 13.64 | 13.61 | 15.41 | 16.70 |  |
| 150 | 13.67 | 13.53 | 15.44 | 16.89 |  |
| 200 | 13.72 | 13.61 | 15.54 | 17.14 |  |
| 250 | 13.84 | 13.55 | 15.58 | 17.03 |  |
| 300 | 13.67 | 13.58 | 15.56 | 17.04 |  |
| 350 | 13.71 | 13.56 | 15.68 | 16.98 |  |
| 400 | 13.66 | 13.58 | 15.49 | 17.09 |  |
| 450 | 13.69 | 13.61 | 15.50 | 17.01 |  |
| 500 | 13.80 | 13.58 | 15.58 | 16.99 |  |
| 550 | 13.82 | 13.58 | 15.75 | 17.09 |  |
| 600 | 13.56 | 13.60 | 15.59 | 17.23 |  |
| 650 | 13.83 | 13.54 | 15.86 | 17.06 |  |
| 700 | 13.58 | 13.55 | 15.65 | 17.03 |  |
| 750 | 13.79 | 13.61 | 15.65 | 17.03 |  |
| 800 | 13.83 | 13.66 | 15.59 | 17.16 |  |
| 850 | 13.89 | 13.59 | 15.45 | 17.03 |  |
| 900 | 13.79 | 13.61 | 15.37 | 17.09 |  |
| 950 | 13.77 | 13.56 | 15.51 | 17.07 |  |
| 1000 | 13.67 | 13.53 | 15.46 | 17.04 |  |
| Average | 13.75 | 13.59 | 15.55 | 16.99 |  |
