# Supplementary material for: Sodium Montmorillonite/Amine-Containing Drugs Complexes: New Insights on Intercalated Drugs Arrangement into Layered Carrier Material
Source: PLoS One. 2015 Mar 24;10(3):e0121110. doi: 10.1371/journal.pone.0121110 (PMC4372448; doi:10.1371/journal.pone.0121110)
Supplement: S6 Table — (DOCX) [file pone.0121110.s008.docx]

**Table S6. Basal spacing (Å) of Na-MMT/NTT models every 50 ps along 1ns of molecular dynamics simulation.**

| **Time** | **Basal spacing (Å)** | | | |  |
| --- | --- | --- | --- | --- | --- |
|  | **Na-MMT/NTT1** | **Na-MMT/NTT2** | **Na-MMT/NTT3** | **Na-MMT/NTT4** |  |
| 0 | 16.03 | 20.19 | 27.26 | 30.97 |  |
| 50 | 16.48 | 21.11 | 27.00 | 31.45 |  |
| 100 | 16.59 | 20.99 | 27.43 | 30.87 |  |
| 150 | 16.59 | 20.62 | 27.41 | 30.84 |  |
| 200 | 16.37 | 20.98 | 27.55 | 30.97 |  |
| 250 | 16.60 | 20.58 | 27.25 | 30.86 |  |
| 300 | 16.53 | 20.89 | 27.54 | 30.90 |  |
| 350 | 16.65 | 20.80 | 27.54 | 31.21 |  |
| 400 | 16.58 | 20.76 | 27.13 | 31.20 |  |
| 450 | 16.55 | 20.85 | 27.65 | 31.31 |  |
| 500 | 16.48 | 20.86 | 27.78 | 31.01 |  |
| 550 | 16.57 | 20.57 | 27.20 | 31.06 |  |
| 600 | 16.56 | 20.68 | 27.24 | 31.21 |  |
| 650 | 16.58 | 20.78 | 27.65 | 31.48 |  |
| 700 | 16.38 | 20.74 | 27.26 | 31.42 |  |
| 750 | 16.46 | 20.78 | 27.29 | 31.62 |  |
| 800 | 16.52 | 21.05 | 27.20 | 31.83 |  |
| 850 | 16.67 | 20.84 | 27.17 | 31.58 |  |
| 900 | 16.54 | 20.78 | 27.07 | 31.37 |  |
| 950 | 16.59 | 20.69 | 27.59 | 31.76 |  |
| 1000 | 16.51 | 20.75 | 27.17 | 31.96 |  |
| Average | 16.51 | 20.77 | 27.35 | 31.28 |  |
